# Tunable Unidirectional Guided Resonances via Multi‐Singularity Interactions in Momentum Space

**DOI:** 10.1002/nap2.70248

**Published:** 2026-08-10

**Authors:** Ying Yu, Siyi Han, Zhiyuan Gu, Dan Wang, Yu Li, Qiao Jiang

**Affiliations:** ^1^ College of Physics and Optoelectronics Taiyuan University of Technology Taiyuan China; ^2^ Guangdong Provincial Key Laboratory of Nanophotonic Manipulation Jinan University Guangzhou China; ^3^ College of Optoelectronic Engineering Chongqing University Chongqing China

**Keywords:** bound states in the continuum, circularly polarized states, topological singularity, unidirectional guided resonances, unidirectional radiation

## Abstract

The topological nature of bound states in the continuum (BICs) has attracted significant interest in manipulating resonant states and light radiation through topological singularities in momentum space. In this work, we present a scheme to realize tunable unidirectional guided resonances (UGRs) based on interactions between multiple circularly polarized states (C points) in momentum space. We first demonstrate that C points can intrinsically exist around symmetry‐protected BICs in photonic crystal slabs even without any symmetry reduction. Through controlled symmetry breaking, the BIC splits into a pair of C points, which can interact with the intrinsic ones. Their interactions lead to rich evolution processes of C points, including formation of two UGRs at different ***k*** points with opposite topological charges, and generation of two UGRs with identical charges accompanied by the annihilation and regeneration of C points. Furthermore, it is demonstrated that directional radiation with high asymmetry ratio can be achieved across a broad parameter range via the interactions between multiple C points. These findings suggest that the involvement of multiple singularities provides additional degrees of freedom for resonant mode manipulation, opening new avenues for investigating singularity dynamics in momentum space and applying unidirectional guided resonances in optical field control.

## Graphical abstract

C points can intrinsically coexist around symmetry‐protected BICs without symmetry reduction. Symmetry breaking splits SP‐BICs into new C points that interact with intrinsic ones, forming perfect UGRs at distinct ***k*** points with tunable topological charges. A band of quasi‐UGRs is further generated across extensive parameter ranges via interactions between multiple C points, enabling new possibilities for robust directional light manipulation.

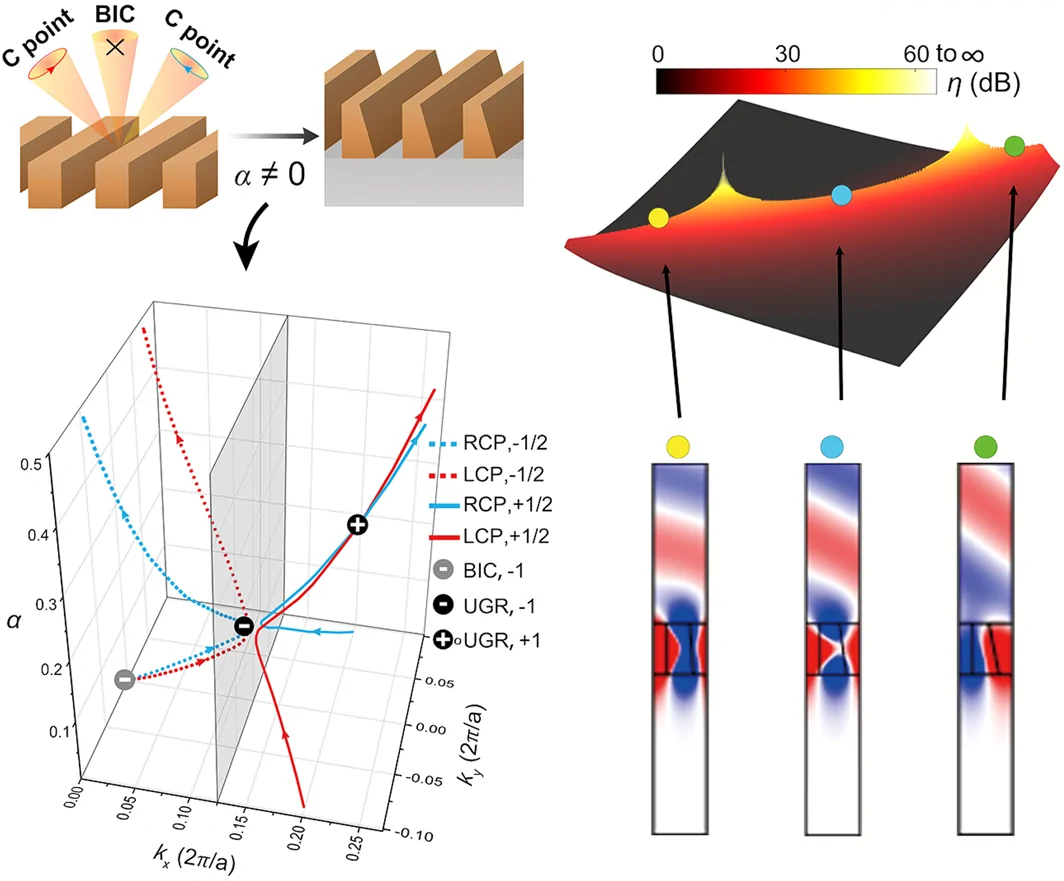

## 1 Introduction

Controlling the propagation of light and achieving unidirectional radiation are critical for a wide range of optical applications, including integrated photonics, light sources, and optical communication. With advancements in nanofabrication techniques, various photonic nanostructures have been proposed to achieve direction control of light in diverse scenarios, such as unidirectional nanoscale photon sources by coupling quantum dots to a nanoantenna [1, 2], efficient unidirectional excitation of surface plasmon polaritons [3, 4, 5], and steering radiation direction of integrated lasers [6]. For planar devices, conventional approaches to achieve directional emission typically rely on reflectors such as metal films or Bragg mirrors. However, these methods either introduce ohmic losses or require multilayer structures, increasing device complexity and thickness. Thus, developing an alternative mechanism to realize unidirectional radiation in a single‐layer dielectric structure is of significant practical importance.

The emergence of bound states in the continuum (BICs) in photonic crystal (PhC) slabs has opened new avenues for manipulating the radiation of light field. BICs are guided modes that lie within the radiation continuum but remain decoupled from free‐space waves [7, 8, 9, 10, 11]. This remarkable property enables the realization of optical resonances with ultra‐high Q factors in planar photonic structures [12, 13, 14], making BICs particularly valuable for enhancing light–matter interactions and finding applications in areas from enhanced optical nonlinearity [15, 16, 17, 18, 19], ultra‐sensitive biological and chemical sensing [20, 21, 22, 23], to low‐threshold nanolasers [24, 25, 26, 27]. Beyond the exceptional resonant properties, recent studies have revealed the topological nature of BICs, which correspond to vortex polarization singularities (V points) of far‐field radiation in momentum space [8, 28, 29]. This insight has led to investigations into the topological characteristics of BICs, including their stability under parameter perturbations, dynamic evolution processes such as merging and annihilation of BICs [8, 30, 31, 32], as well as novel applications in structured light [33]. Moreover, by introducing structural symmetry breaking, a BIC can be split into a pair of circularly polarized states (C points) with identical topological charges but opposite handedness, which has offered new opportunities for realizing novel chiral optical effects including chiral BIC, chiral emission, and tunable chiroptical response [34, 35, 36, 37, 38, 39]. In recent studies, diverse modulation phenomena of polarization singularities in momentum space have been realized by introducing material anisotropy and magneto‐optical responses, which further demonstrate the broad application prospects of topological properties of guided resonances for optical field manipulation [40, 41, 42, 43].

Inspired by the nonradiative and topological properties of BICs, a new mechanism has been proposed to achieve unidirectional guided resonances (UGRs), where light emission is restricted to one side of the PhC slab [44]. The formation of UGRs involves two key steps: (1) obtaining a pair of half‐charged C points with opposite handedness; (2) carefully tuning the asymmetry parameters of the PhC slab to merge these C points into a V point in the far field radiation on one side, consequently suppressing radiation in that direction. For instance, by breaking the in‐plane inversion (C_2_) symmetry of a PhC slab, an accidental BIC splits into a pair of C points. Further breaking the up‐down mirror symmetry induces inequivalent evolutions of C points in upward and downward radiation channels, enabling the formation of a UGR. For the downward radiation, when the structure is continuously varied, the C points can evolve in momentum space until they bounce into each other and become a V point, forbidding downward radiation from the structure and only allowing upward radiation (i.e., forming a UGR). Relevant studies also demonstrate that the emission angle of UGRs can be flexibly controlled by adjusting the geometric parameters of the structure [45, 46, 47]. Further research studies show that C‐point pairs need not originate solely from BICs; they can also emerge from band degeneracies or zero topological charge in a two‐layer PhC slab [46, 48, 49]. However, these previous UGR designs rely on C‐point pairs from a single origin (e.g., by breaking a BIC or from band degeneracies), requiring precise geometric control of PhC slabs and offering limited adjustability.

In this work, we demonstrate tunable UGRs in a one‐dimensional PhC slab by controlling interactions between multiple C points—namely the evolution and collision of a pair of intrinsic C points and another pair of C points split from a symmetry‐protected BIC (SP‐BIC). We first reveal that C points can exist around a SP‐BIC at the *Γ* point in a photonic crystal slab preserving both in‐plane *C*_2_ and up‐down mirror symmetries. Notably, we call these C points as intrinsic C points, as they arise without artificial structural symmetry breaking, which differs from the induced C points reported in previous studies that are generated by deliberate symmetry‐breaking perturbations. By introducing symmetry breaking, the SP‐BIC splits into C points and can interact with the intrinsic ones. By varying the asymmetry parameter and thickness of the structure, we observe rich evolution processes of C points, including (1) formation of two UGRs at distinct ***k*** points with opposite topological charges; (2) annihilation and regeneration of C points, which leads to formation of two UGRs with identical charges. Furthermore, we demonstrate that interactions between multiple C points enable unidirectional radiation across broad parameter ranges, in contrast to the stringent geometric parameter requirements in previous designs [44, 46].

## 2 Coexistence of BIC and C Points

As shown in Figure 1a, we first consider a freestanding one‐dimensional PhC slab with both in‐plane *C*_2_ symmetry and up‐down mirror symmetry. The slab consists of periodic silicon bars (refractive index $n_{1}=3.48$) along the *x* axis with lattice constant *a* = 500 nm and width *w* = 250 nm. The thickness of the slab is selected as a tuning parameter. Contrast to previous studies in which the generation of C points requires symmetry breaking to destroy a BIC, our work shows that C points can coexist with the SP‐BIC at *Γ* point ($k_{x}=k_{y}=0$) on a nondegenerate band without reducing the structure symmetry. To verify this, we perform numerical calculations and analysis on the guided resonances supported by the designed PhC slab (see Supporting Information S1: Section S1 for more details of simulation). Here, we focus on the lowest TE‐like band (TE_1_) for a PhC slab of thickness H= 480 nm [see the blue line in Figure 1b). In Figure 1c, the divergent *Q* factor at *Γ* point indicates the presence of a SP‐BIC, which results from the symmetry mismatch between the mode profile inside the PhC slab and the extended propagating modes in free space [7, 8, 50]. The electric field profile in the inset clearly demonstrates the disappearance of leakage radiation from the SP‐BIC.

**FIGURE 1:**
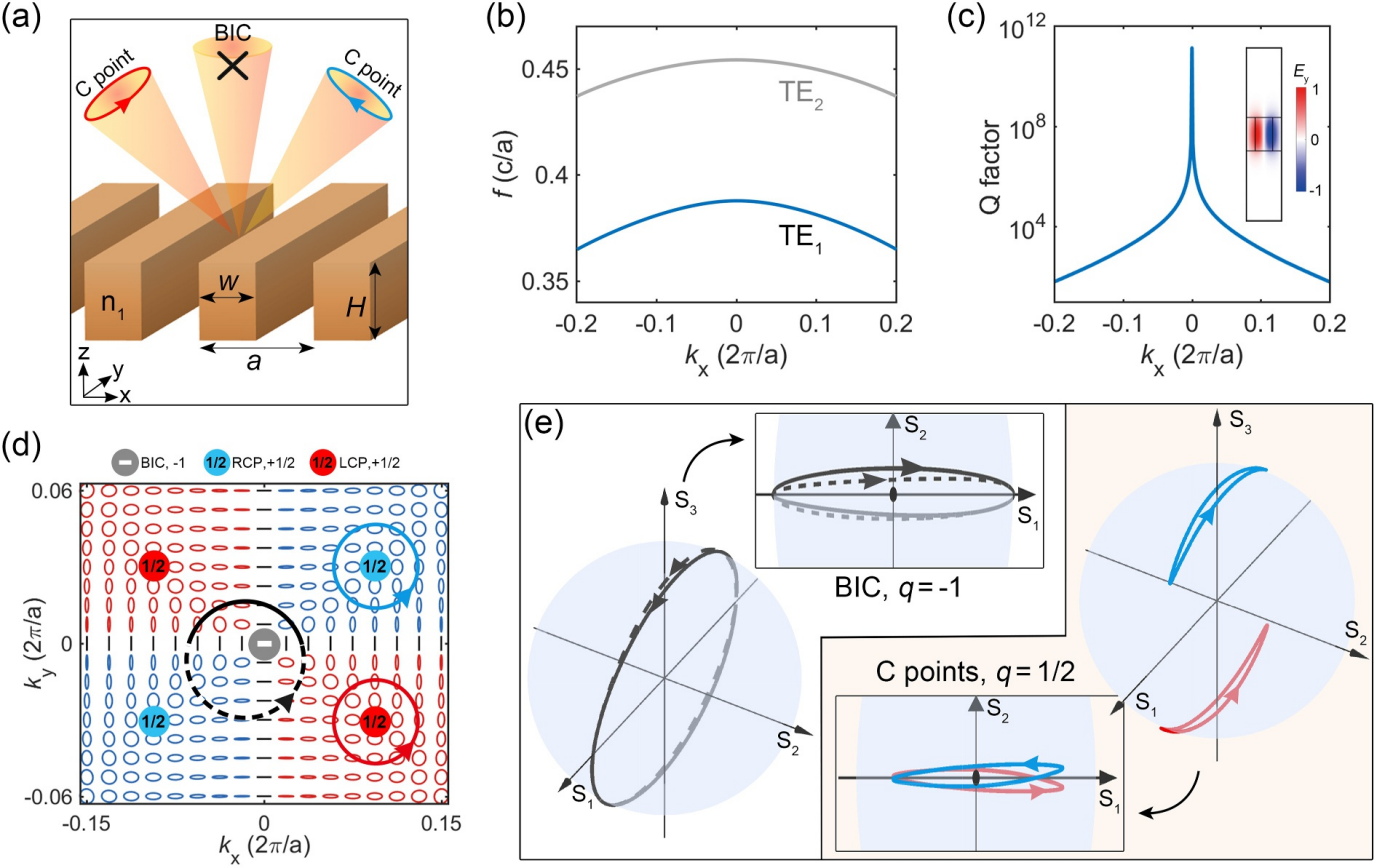
Coexistence of BIC and C points. (a) Schematic of a one‐dimensional PhC slab with in‐plane *C*_2_ symmetry and up‐down mirror symmetry, which supports both the SP‐BIC and C points. (b) Calculated TE‐like band structure along the $k_{x}$ axis. (c) Q factor of the TE_1_ band. The inset shows the electric field profile $E_{y}$ of the guided resonance mode at $k_{x}=0$. (d) Far‐field polarization states distribution of the TE_1_ band. The ellipses of blue (red) color represent right‐ (left‐) handed polarization states with Stokes parameter $S_{3}>0$ ($S_{3}<0$). The BIC and C points are indicated as gray and blue/red (RCP/LCP) dots, respectively. (e) Trajectories of polarization states on the Poincaré sphere corresponding to the anticlockwise close loops in (d).

To further investigate polarization singularities in momentum space, the polarization states of the upward far‐field radiation are plotted in Figure 1d. It can be observed that there are 4 C points (marked by blue and red dots) around the SP‐BIC (the gray dot), two of which exhibit right‐handed circular polarization (RCP, blue dots), whereas the other two are left‐handed (LCP, red dots). Notably, these C points can stably exist under in‐plane $C_{2}$ symmetry and up‐down mirror symmetry rather than arising from accidental parameter settings (see more details in Supporting Information S1: Figure S1). To quantitatively discuss the topological properties of these polarization singularities, the topological charge *q* carried by a singularity can be defined as the winding number of the major axis of polarization states around it [8, 50]:

(1) $$q=\frac{1}{2\pi }\oint_{L}dk_{∥}\nabla_{k_{∥}}\phi \left(k_{∥}\right)$$

where *L* is a counterclockwise closed loop around the singularity in momentum space. $\phi \left(k_{∥}\right)$ is the orientation angle of the polarization states' major axis for far‐field radiation. $k_{∥}=\left(k_{x},k_{y}\right)$ is the in‐plane wave vector.

With the above definition, a BIC (or V point) can be characterized by an integer topological charge, whereas the charge of a C point corresponds to a half integer. To visualize the topological charges, we map the far‐field polarization states along closed loops in Figure 1d onto the Poincaré sphere. As shown in the left part of Figure 1e, the polarization states around the SP‐BIC (see the black loop in Figure 1d) correspond to a trajectory encircling the $S_{3}$ axis twice in clockwise direction, indicating a −1 topological charge of the SP‐BIC. It is worth noting that the trajectory lies very close to the S_1_‐S_3_ plane markedly differs from conventional BIC behavior where polarization states typically remain linear (confined to the equatorial region of Poincaré sphere) [8, 34]. This can be attributed to the presence of the C points, which causes the polarization states around the BIC to deviate from the equator and shift toward the poles of the Poincaré sphere. As for the C points, a topological charge of +1/2 can be determined from the counterclockwise trajectories on the Poincaré sphere for both the RCP and LCP C points (see the right part of Figure 1e). The topological charge can also be effectively calculated numerically from discrete simulated data; see more details in Supporting Information S1: Section S2.

## 3 Tunable UGRs Arising From Interaction of Different Singularities

The coexistence of the SP‐BIC and C points provides additional degrees of freedom to manipulate the radiation from guided resonances by controlling the interaction of different singularities in momentum space. To fully utilize them for controlling the radiation directionality and realizing UGRs, we introduce a variable asymmetry parameter α to tune the evolution of singularities. As shown in Figure 2a, the silicon bar has a fixed bottom width w=250nm and a tunable top width (1−α)w. In consideration of practical implementations, a silica substrate with a refractive index of 1.45 is applied into the design, and the designed structure can be experimentally fabricated via electron‐beam lithography and subsequent slanted etching process [35, 44]. When α≠0, the right sidewall of the silicon bars is tilted to break both the in‐plane *C*_2_ and up‐down mirror symmetries of the PhC slab, causing the SP‐BIC to split into a pair of C points with opposite handedness, which can interact with the intrinsic C points by further increasing the degree of symmetry breaking. Due to the absence of up‐down mirror symmetry, the radiations toward the top and the bottom differ from each other. When continually increasing α, it is found that the C points in the downward radiation can merge together to form V points at different ***k*** points (see Figure 2b), leading to the generation of tunable UGRs.

**FIGURE 2:**
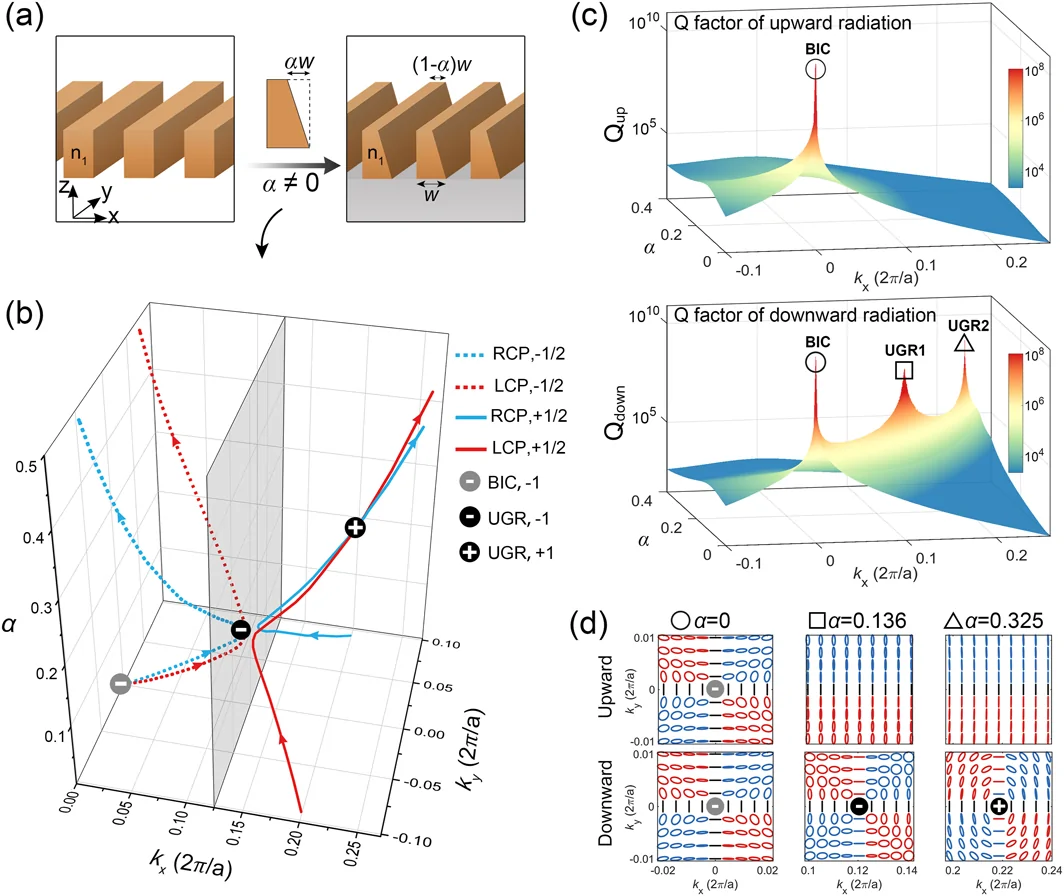
Tunable UGRs arising from a SP‐BIC and two intrinsic C points by symmetry breaking. (a) Schematics of the photonic crystal slab supporting tunable UGRs. The sidewall of the structure is tilted to introduce symmetry breaking, and α is the asymmetric parameter. (b) Evolution of C points in the downward radiation in momentum space by increasing α. The thickness of the slab is H= 480 nm. (c) Q factors of upward and downward radiations when varying α, where the original BIC at *Γ* point and two formed UGRs can be observed from the divergent points. (d) Far‐field polarization states in upward and downward radiation in momentum space around the original BIC (α=0) and two UGRs (α=0.136 and 0.325).

Since the C points in upward radiation do not merge with increasing *α* in our case, to investigate the mechanism of tunable UGRs, we focus on the C point evolution in downward radiation with $k_{x}\geq 0$ (see more details in Supporting Information S1: Figure S2). As shown in Figure 2b, by increasing *α*, the pair of C points (q=−1/2) spawned from the SP‐BIC keeps moving along the dashed trajectories and merge together as a V point (see the black dot, q=−1) at $k_{∥}=\left(0.1206,0\right)2\pi /a$ when *α* is increased to 0.136. Meanwhile, the other pair of C points (intrinsic C points with q=+1/2, see the solid trajectories) moves toward the $k_{x}$ axis ($k_{y}=0$). When we continue to increase *α*, the V point splits into a pair of C points with q=−1/2 again and the other two intrinsic C points will merge together at $k_{∥}=\left(0.2186,0\right)2\pi /a$ for *α* = 0.325, forming another V point of q=+1. Because V points are singularities of polarization, forbidding any radiative loss from the bottom side of PhC slab, the corresponding guided resonances become UGRs that can only radiate upward.

To verify the unidirectional radiation of these UGRs, we plot the upward ($Q_{up}$) and downward ($Q_{\text{down}}$) radiative *Q* factors while varying the asymmetry parameter (Figure 2c). Due to the *y*‐mirror symmetry of the structure, the C points can only collapse on the $k_{x}$ axis. Therefore, in Figure 2c, we focus on the evolution of $Q_{up}$ and $Q_{down}$ along this axis to analyze the far‐field radiation characteristics. It can be observed that there are three divergent points of $Q_{\text{down}}$ [see the lower panel in Figure 2c), corresponding to three guided resonances that cannot radiate to the bottom of the structure. Comparing $Q_{up}$ and $Q_{\text{down}}$, the first divergent point (marked by a circle) at $k_{x}=0$ for α=0 is obviously the SP‐BIC, where both upward and downward radiations vanish. As for the other two divergent points of $Q_{\text{down}}$ (marked by a square and triangle, respectively), it is evident that the corresponding $Q_{\text{up}}$ values of these modes remain finite, confirming the existence of upward radiation. In other words, these resonant states are exactly the UGR modes. Here, we label them as UGR1 and UGR2, respectively. In Figure 2d, we plot the polarization states of upward and downward radiations around the BIC, UGR1, and UGR2 for α=0, 0.136, and 0.325. It can be seen that V points exist in both the upward and downward radiations for α=0, conforming to divergent $Q_{\text{up}}$ and $Q_{\text{down}}$ of the BIC. For UGR1 and UGR2, V points only exist in the downward radiations, carrying topological charges −1 and +1 [see the middle and right panels of Figure 2d), which agrees with the process in Figure 2b and clearly demonstrates the distinct topological nature of these UGR modes.

To give an insight into the formation mechanism of these UGRs in momentum space. In Figure 3a,d, we calculate and plot the far‐field polarization states of both upward and downward radiations for different α, where the projected evolution trajectories of C points are added for eye guidance (see the dashed and solid lines). As there are no C points in the upward radiation, we focus on the evolution of C points in the downward radiations. As shown in Figure 3a, when α is increased from 0.104 to 0.15, it can be seen that the 2 C points of q=−1/2 (generated from the SP‐BIC) move toward each other and merge on the $k_{x}$ axis to form a V point (UGR1) of q=−1 (α=0.136), which will split into a pair of C points again (α=0.15). To quantitatively study the unidirectional radiation, we define an asymmetry ratio between upward and downward radiation, $\eta =Q_{\text{down}}/Q_{\text{up}}$, for different ***k*** points (see more details in Supporting Information S1: Section S3). A divergent point of η in Figure 3b further verifies the formation of UGR1 at $k_{∥}=\left(0.1206,0\right)2\pi /a$, which can only radiate upward (see Figure 3c).

**FIGURE 3:**
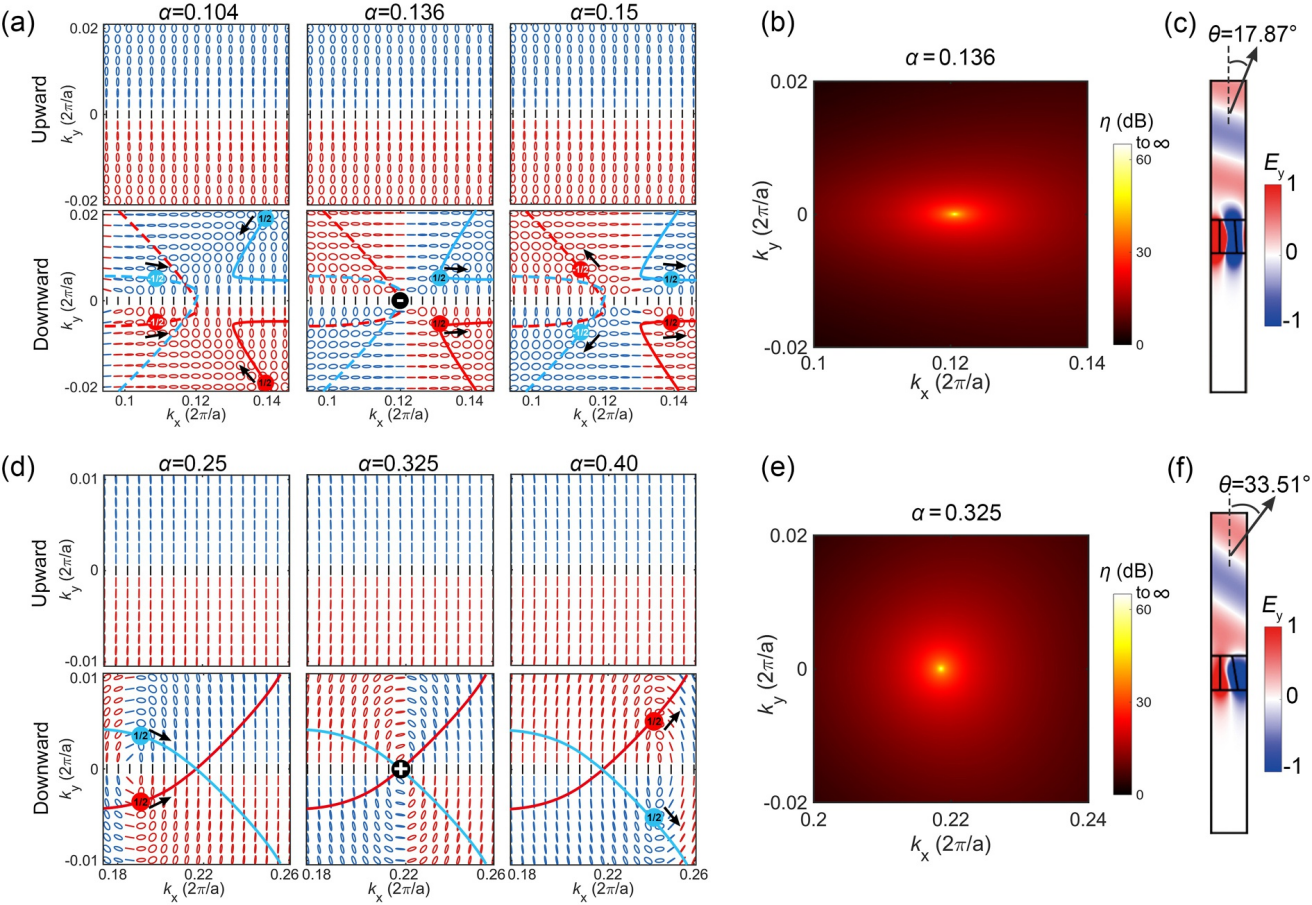
Formation of tunable UGRs with opposite topological charges. (a) Far‐field polarization states of upward and downward radiations in momentum space for α=0.104, 0.136, and 0.15. UGR1 of =−1. (b) The asymmetry ratio η between upward and downward radiation for α=0.136. (c) The electric field profile $E_{y}$ of UGR1 mode at $k_{∥}=\left(0.1206,0\right)2\pi /a$. (d) Far‐field polarization states for α=0.25, 0.325 and 0.40. (e) The asymmetry ratio η for α=0.325. (f) The electric field profile $E_{y}$ of UGR2 mode at $k_{∥}=\left(0.2186,0\right)2\pi /a$.

When we continue to increase α from 0.25 to 0.40, Figure 3d exhibits that the two intrinsic C points of q=+1/2 gradually move close to the $k_{x}$ axis and merge together into a V point of q=+1 (UGR2) when α=0.325. This can be clearly verified by the divergent point of the asymmetry ratio η in Figure 3e and unidirectional radiation profile in Figure 3f. Similar to the case of UGR1, UGR2 also splits into a pair of C points again when continuing to increase α (see right panels in Figure 3d). The evolution processes in Figure 3a,d conform the formation mechanism of UGRs with tunable topological charges shown in Figure 2b. Moreover, as the two UGRs are formed at different ***k*** points, it is allowed to realize unidirectional radiations with tunable emission angles. Here, the angles of unidirectional emission can be changed from 17.87° (UGR1) to 33.51° (UGR2) (see Figure 3c,f). This feature can be applied in angle‐dependent photonic devices such as directional light sources and grating coupler.

Interestingly, in the above processes, although the 2 C points generated from the SP‐BIC do not collide with the two intrinsic ones, it can be observed that they gradually moved closer to each other. And the two intrinsic C points subsequently change direction and move away along $+k_{x}$ direction after UGR1 splits into C points. This phenomenon implies the interactions between these C points. To regulate the interactions, we decrease the structure thickness to 475 nm and plot the evolution trajectories of C points in Figure 4a, which shows a completely different evolution process compared to that in Figure 2b. It can be clearly observed that the entire evolution process consists of two parts (separated by a gray plane in Figure 4a).

**FIGURE 4:**
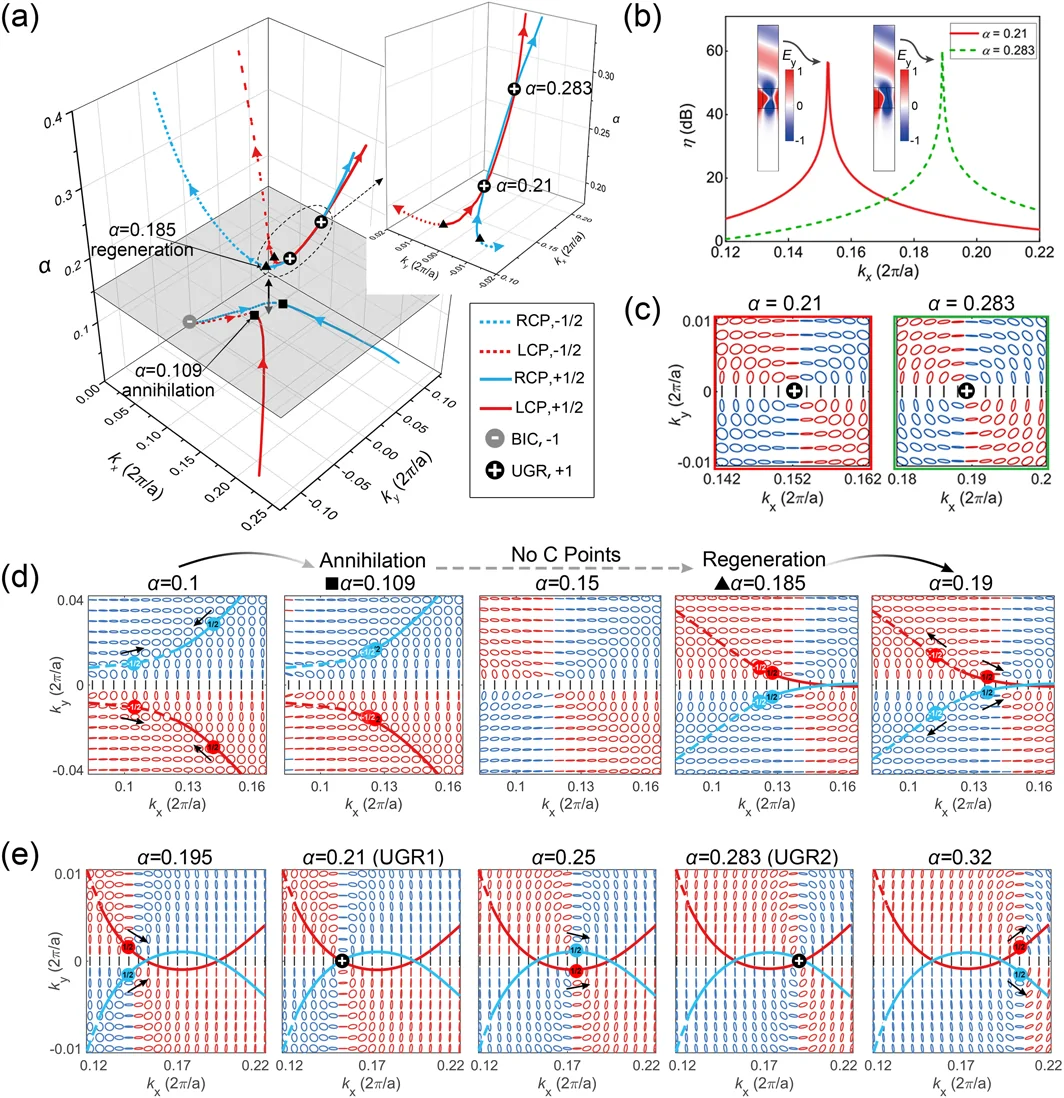
Regulate the interaction of multiple C points to generate two UGRs with the same topological charges. (a) Evolution of C points for a PhC slab of thickness H=475nm when continuously increasing *α*. (b) The asymmetry ratio along the $k_{x}$ axis for α=0.21and0.283. The insets show the electric field profile $E_{y}$ of the two UGR modes corresponding to the divergent points of η. (c) Far‐field polarization states of downward radiation for α=0.21 (the left panel) and 0.283 (the right panel). (d) Polarization states of downward radiation for α=0.1 to 0.19, showing the annihilation (α=0.109) and regeneration (α=0.185) of two pairs of C points. (e) Polarization states of downward radiation from α=0.195 to 0.32, showing the formation of two UGRs with the same topological charge +1 at α=0.21 (UGR1) and α=0.283 (UGR2).

In the first part (see the red and blue lines below the gray plane in Figure 4a), as *α* is gradually increased from 0, two pairs of C points gradually approach each other. At *α* = 0.109, C points of the same handedness but opposite topological charges collide and annihilate each other (marked by black squares). In the second part (see the red and blue lines above the gray plane), when *α* increases to about 0.185, two new pairs of C points are generated again (marked by black triangles). As we continue to increase *α*, the 2 C points of q=−1/2 (see the red and blue dashed lines) move toward ‐*k*_*x*_ direction and gradually separate from each other, whereas the other pair of C points of q=+1/2 (red and blue solid lines) merges twice on the *k*_*x*_ axis, forming two UGRs at different *k*_*x*_ positions (see the black dots in Figure 4a). In Figure 4b, the divergent asymmetry ratio at $k_{∥}=\left(0.1526,0\right)2\pi /a$ and (0.1891,0)2π/a verifies the existence of two UGRs for α=0.21 and 0.283, respectively. The electric‐field profiles in Figure 4b further prove the unidirectional radiation of these UGRs. It is worth noting that since these two UGRs are generated by the merging of the same pair of C points with +1/2 charges, their corresponding topological charges are both +1 according to the conservation law of topological charge (see Figure 4c).

For a better understanding of the interactions between these C points, in Figure 4d,e, we plot the polarization states of downward radiation from α=0.1 to 0.32. As shown in Figure 4d, it can be seen that C points with opposite topological charges and the same handedness approach to each other when α is increased from 0.1 to 0.109 (see the black arrows for eye guidance), and they finally collide to annihilate each other. As α is further increased, no C points appear until it reaches 0.185, at which point two new pairs of C points are generated again. This process corresponds to the gap between the evolution trajectories in Figure 4a (marked by the black double‐headed arrow). When we continue to increase α from 0.185 to 0.19, the polarization states show that the pair of C points with charge of +1/2 (see red and blue solid lines) gets closer but the other two with charge of −1/2 (see red and blue dashed lines) move away from each other. In Figure 4e, we focused on the 2 C points of q=+1/2 and gradually increase α from 0.195 to 0.32. At α=0.21, the 2 C points cross into each other and form a V point of q=+1 at $k_{∥}=\left(0.1526,0\right)2\pi /a$, resulting in the disappearance of downward radiation (i.e., forming UGR1). When α increases from 0.21 to 0.283, 2 C points with the same charge q=+1/2 are spawned from the V point and merge again at $k_{∥}=\left(0.1891,0\right)2\pi /a$, forming another UGR mode (UGR2).

## 4 Unidirectional Radiation From Quasi‐UGRs

As mentioned above, we demonstrate that the interactions between multiple C points can be controlled by varying the PhC slab thickness, leading to distinct formation mechanisms of tunable UGRs at different ***k*** points. Although perfect UGRs exhibit purely upward radiation due to the forbidden topological effect (where the radiation asymmetry ratio *η* diverges to infinity), the interactions between multiple C points can give rise to quasi‐UGRs (i.e., guided resonances with finite but fairly high *η*) across a broad parameter range. To illustrate this, we map the asymmetry ratio *η* in *k*_x_‐*α* space for slab thicknesses of *H* = 480 nm, 475 nm, and 470 nm in Figure 5a, where a pronounced high‐*η* band is evident. For *H* = 480 and 475 nm, two divergent points of *η* appear, corresponding to the two UGRs. In contrast, no UGRs are observed for *H* = 470 nm because the two regenerated C points of *q* = +1/2 fail to merge as V points on the *k*_*x*_ axis when changing α (see the schematic of C point evolution in the inset).

**FIGURE 5:**
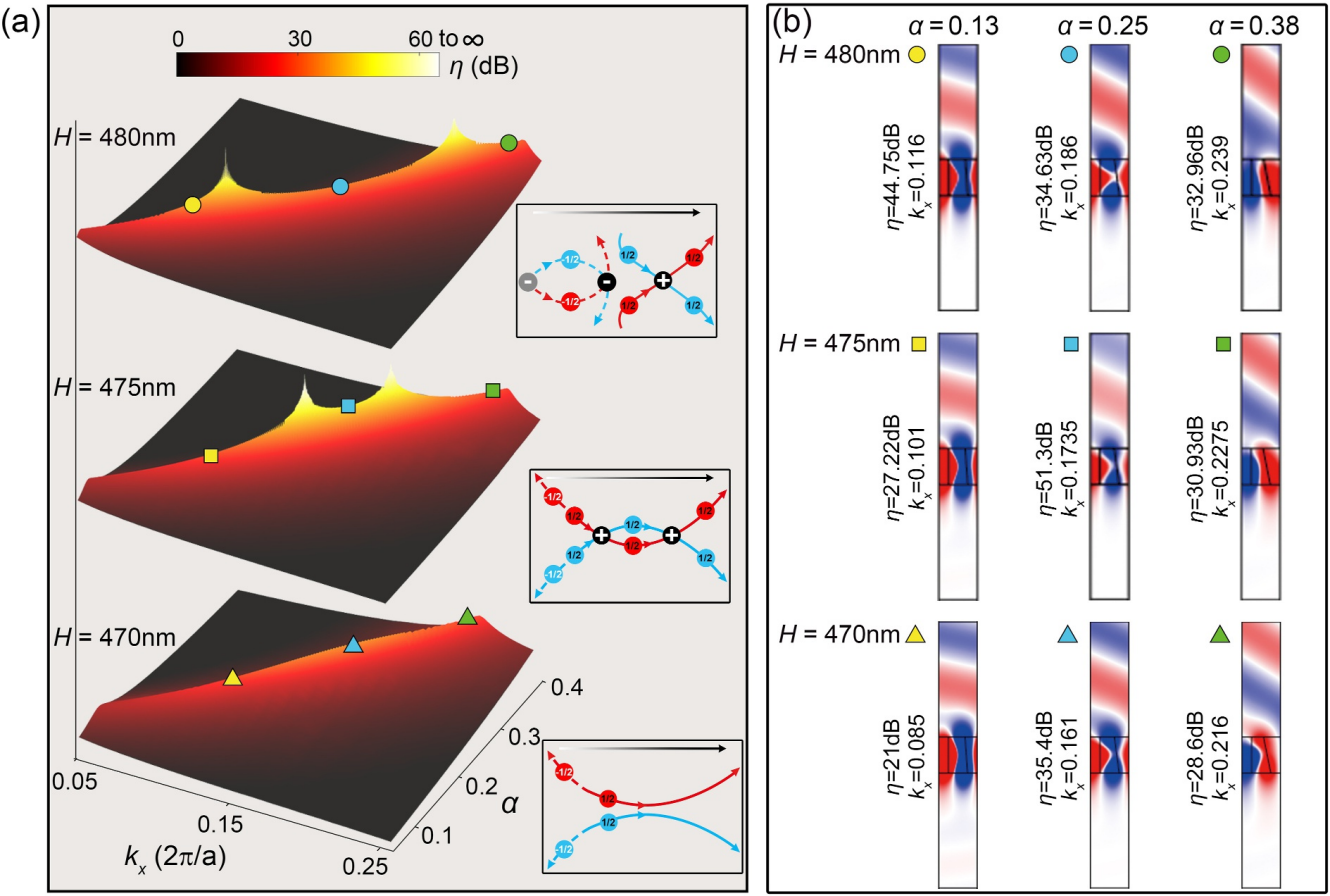
Unidirectional radiation from quasi‐UGRs across a wide parameter range. (a) Evolution of the asymmetry ratio η when increasing α for PhC slabs with different thicknesses. The insets illustrate the corresponding evolution processes of C points. (b) Electric field profile of the modes at α=0.13,0.25,and0.38 for different thicknesses, which are accordingly marked with circles, squares, and triangles of different colors in (a).

Here, we focus on the modes with finite but large *η* on these high‐*η* bands, and we define a mode as a quasi‐UGR if its *η* > 20 dB; these quasi‐UGRs exhibit strong unidirectional radiation performance. In Figure 5b, we plot the electric‐field profiles of the quasi‐UGR modes for α=0.13,0.25, and 0.38 (marked by yellow, blue, and green symbols in Figure 5a), which show that all these modes mainly radiate upward with a large η above 20 dB. These results indicate that the multiple C point interaction provides an effective approach to achieve guided resonances with high unidirectional radiation performance. In practical scenarios, guided resonances are sensitive to structural parameters. Accordingly, the performance of photonic crystal slabs is inevitably affected by fabrication errors, disordered defects, and finite‐size effects [12, 51]. The proposed quasi‐UGR can relax the stringent requirement on structural parameter accuracy to a certain extent and allows effective directional radiation, which endows the design with promising prospects for practical photonic device applications (see Supporting Information S1: Figures S3–S5).

## 5 Conclusion

In summary, we have proposed an approach for achieving and controlling unidirectional radiation in PhC slabs by exploiting interactions between multiple C points in momentum space. Our study first demonstrates that C points can intrinsically coexist around a SP‐BIC without reducing the structure symmetry, providing additional degrees of freedom for manipulating the interactions between multiple polarization singularities. Through controlled symmetry breaking, the intrinsic C points interact with those spawned from the SP‐BIC, enabling the creation of perfect UGRs at distinct ***k*** points. It is worth noting that the topological charge of UGRs can also be tailored, which is unattainable in systems involving only one pair of C points [44]. It is further shown that precise control of these interactions can be achieved by varying the PhC slab thickness, leading to rich C point evolution dynamics in momentum space. This have revealed a new mechanism for tuning the topological nature of UGRs through annihilation and regeneration of C point pairs. Moreover, beyond the tunability of position and topological charges of perfect UGRs, the interactions between multiple C points enable a high‐*η* band of quasi‐UGRs exhibiting robust unidirectional radiation across extensive parameter ranges, substantially relaxing the need for precise structural parameter control in fabrication. Our findings not only reveal the potential of achieving richer manipulation of resonance mode radiation properties by involving interactions of multiple singularities but also opens new avenues for robust and tunable directional light control in compact dielectric nanostructures, which may find applications in integrated photonic devices such as grating couplers and angle‐selective light sources.

## Author Contributions

**Ying Yu:** conceptualization, methodology, writing – review and editing, funding acquisition. **Siyi Han:** methodology, software, investigation, visualization. **Zhiyuan Gu:** validation, methodology, writing – review and editing. **Dan Wang:** writing – review and editing, funding acquisition, validation. **Yu Li:** validation, funding acquisition, writing – review and editing. **Qiao Jiang:** conceptualization, methodology, investigation, validation, funding acquisition, writing – original draft, writing – review and editing.

## Funding

This work is supported by the National Natural Science Foundation of China (Grant Nos. 12474314, 12104333, 62575203), Fundamental Research Program of Shanxi Province (Grant No. 202403021211155), and Open Fund of Guangdong Provincial Key Laboratory of Nanophotonic Manipulation (Grant No. 2023B1212010008), China Postdoctoral Science Foundation (Grant No. 2025M783423), and the Key Laboratory of Cleaner Intelligent Control on Coal & Electricity, Ministry of Education, P. R. China (Grant Nos. CICCE202518, CICCE202519).

## Conflicts of Interest

The authors declare no conflicts of interest.

## Supporting information


Supporting Information S1

## Data Availability

The data that support the findings of this study are available from the corresponding author upon reasonable request.
