# Supplementary material for: Tunable Unidirectional Guided Resonances via Multi‐Singularity Interactions in Momentum Space
Source: Nanophotonics. 2026 Aug 10;15(15):e70248. doi: 10.1002/nap2.70248 (PMC13642075; doi:10.1002/nap2.70248)
Supplement: Supplementary file 1 [file NAP2-15-e70248-s001.pdf]

Supporting Information

Tunable unidirectional guided resonances via multi-singularity interactions in momentum space

Ying Yu, Siyi Han, Zhiyuan Gu, Dan Wang, Yu Li*, and Qiao Jiang**

S1. Numerical simulations of the photonic crystal slab

All numerical simulations in the main text are performed using the finite element method (FEM). A two-dimensional model is employed to investigate the one-dimensional photonic crystal slab. Periodic boundary conditions are applied along the periodic *x* direction, and perfectly matched layers (PMLs) are utilized along the out-of-plane *z* direction. Silicon with a refractive index of 3.48 is selected as the slab material. Mesh convergence tests are carried out by gradually refining the mesh size of the computational domain until the eigenfrequencies of guided resonances converge, and subsequent calculations are conducted with the converged mesh. We solve the eigenmodes of the structure and extract the leakage fields of guided resonances at the upper and lower boundaries for further analysis.

S2. Numerical calculation of the topological charge of a polarization singularity

According to the definition of the topological charge Q of a polarization singularity, the topological charge can be numerically evaluated via:

$$Q = \frac{\Delta\phi}{2\pi}$$

where $\Delta\phi$ denotes the total variation of the orientation angle ϕ of the polarization ellipse's major axis along a counterclockwise closed loop encircling the singularity. Suppose the closed contour is discretized into N sampling points ranging from the azimuthal angle 0 to 2π, yielding polarization angles $\phi_0, \phi_1, \phi_2 \dots, \phi_{N-1}$. The angular difference between two adjacent points is defined as $\delta\phi_i = \phi_i - \phi_{i-1}$. Considering the π-periodicity of ϕ, any angular jump exceeding

$\pi/2$ corresponds to an artificial discontinuity and requires unwrapping correction. We implement the following unwrapping criterion:

$$\delta\phi_i' = \begin{cases} \delta\phi_i - \pi, & \delta\phi_i > \pi/2 \\ \delta\phi_i + \pi, & \delta\phi_i < -\pi/2 \\ \delta\phi_i, & |\delta\phi_i| \le \pi/2 \end{cases}$$

The total variation of the polarization angle is then $\Delta\phi = \sum_{i=1}^{N-1} \delta\phi_i'$, and the topological charge is calculated as $Q = \Delta\phi/2\pi$.

S3. Definition and calculation of radiation asymmetry ratio

In general, the far-field radiation directionality is commonly characterized by the ratio of radiative decay rates $\eta = \gamma_{\text{up}}/\gamma_{down}$. Considering the relation between radiative decay rate and radiation quality factor $\gamma_{\text{up}} = \omega/\text{Q}_{\text{up}}$ ($\gamma_{\text{down}} = \omega/\text{Q}_{\text{down}}$), it can be derived that $\eta = \gamma_{\text{up}}/\gamma_{down} = \text{Q}_{\text{down}}/\text{Q}_{up}$. Therefore, in the main text, we define the radiation asymmetry ratio $\eta = \text{Q}_{\text{down}}/\text{Q}_{up}$, which is physically equivalent to the conventional decay rate ratio.

The radiative Q factor is defined as: $Q = \frac{W*\omega}{P}$, where W denotes the total electromagnetic energy stored inside the photonic crystal slab, and P represents the total outward radiation power. Accordingly, the directional radiative Q factors (Q_{up} and Q_{down}) are calculated by the formula:

$$Q_{up/down} = \frac{W*\omega}{P_{up/down}}$$

Herein, P_{up} and P_{down} stand for the upward and downward radiated power, which are separately obtained by integrating the Poynting vector on the upper and lower sides of the slab. In this way, these two directional Q factors can be effectively separate and extract.

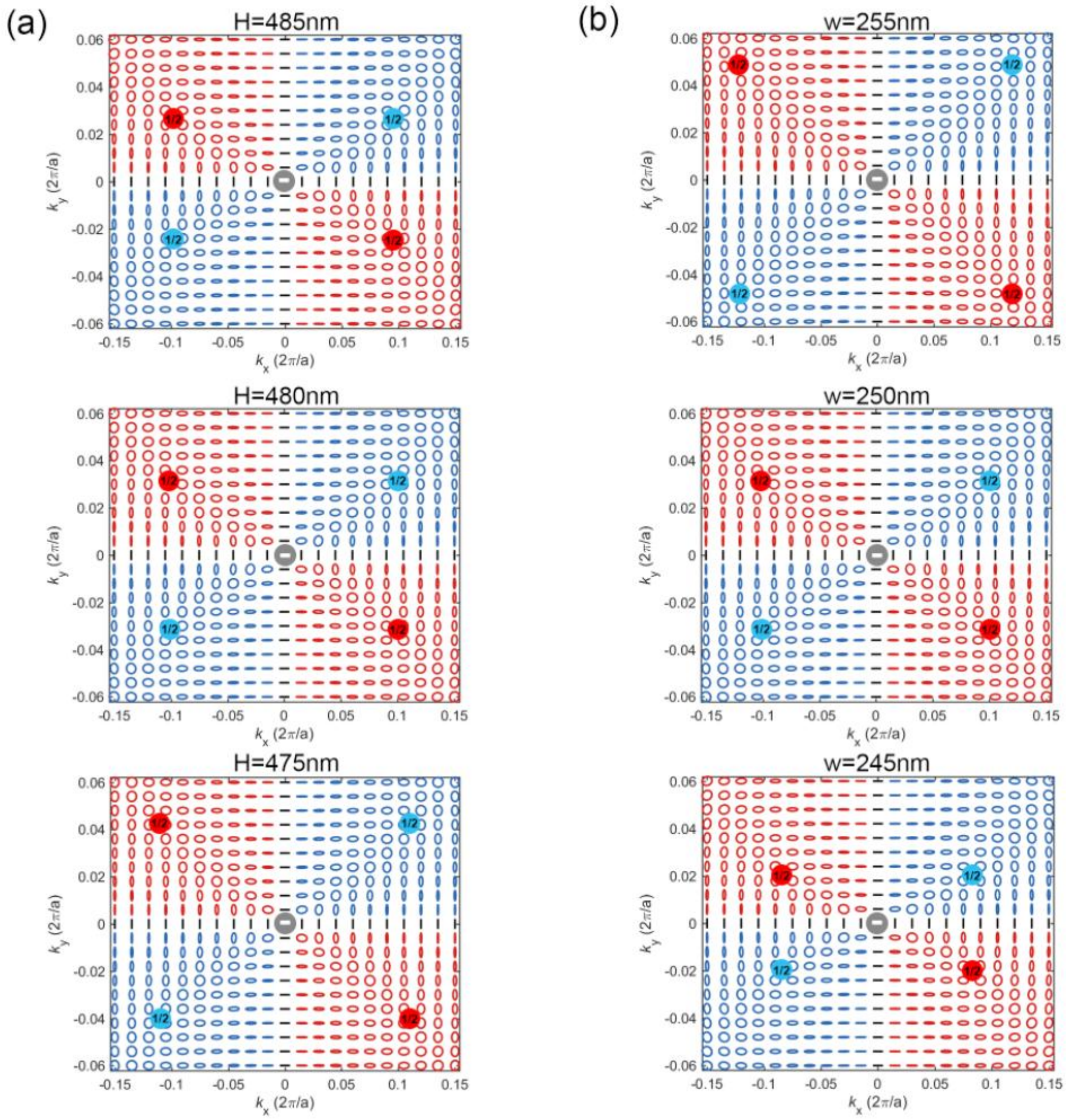

Figure S1 (a) Far-field polarization states distribution for different slab thickness from H = 475 to 485 nm with a fixed width *w* = 250 nm. (b) Far-field polarization states distribution for different width from *w* = 245 to 255 nm with a fixed thickness H = 480 nm. All the other parameters are the same with the structure in Figure 1 of the main text. These results reveal that C points always coexist with the SP-BIC, with only slight changes in their relative positions in momentum space.

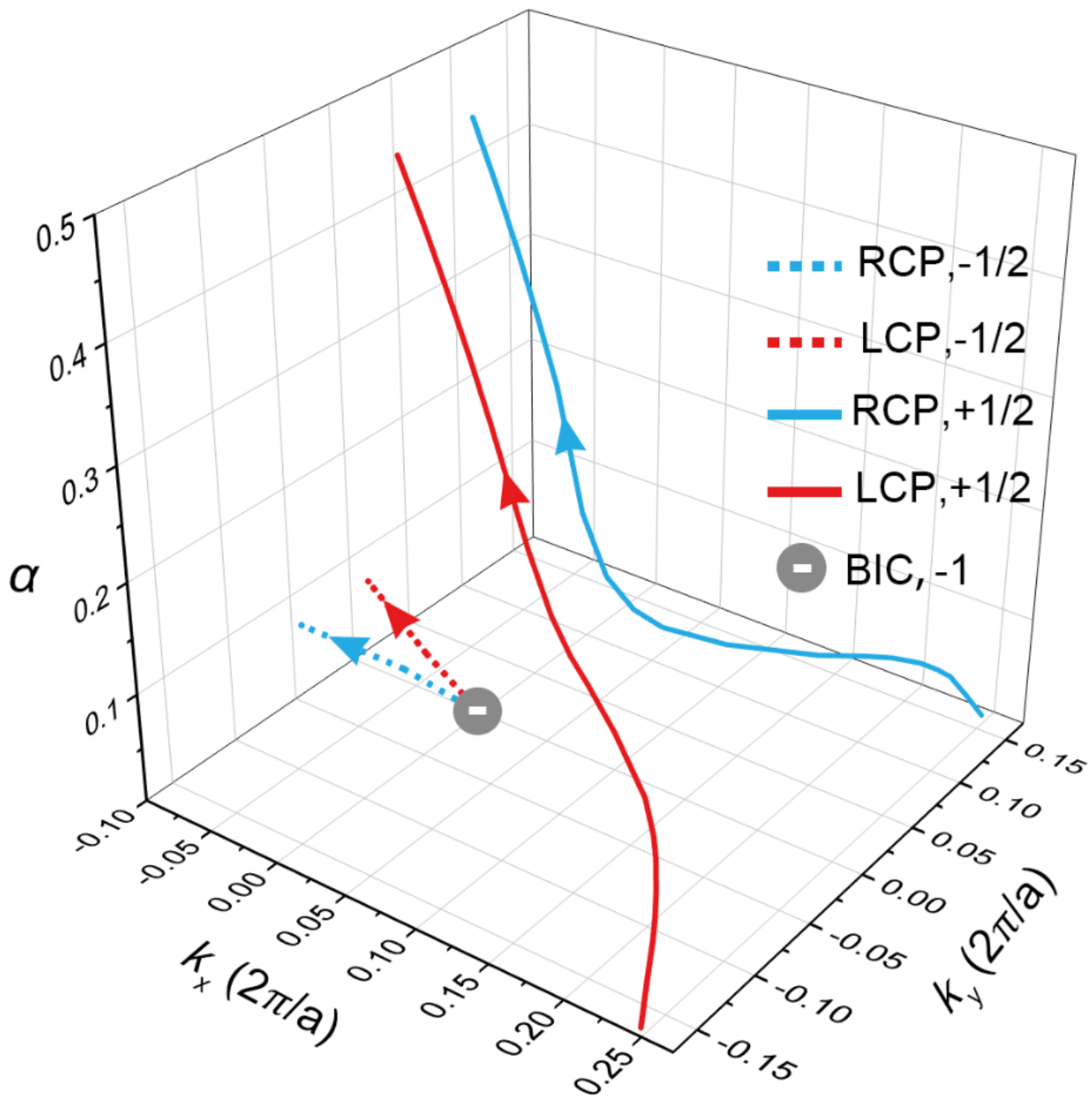

Figure S2 Evolution of C points in the upward radiation in momentum space by increasing α, corresponding to the case of downward radiation in Fig. 2(b) of the main text. It can be observed that the C points generated from the SP-BIC and the intrinsic C points both move along the -k_x direction without merging on the k_x axis throughout the process. This result coincides with the upward Q factor in Fig. 2(c) in the main text, which shows no divergence of Q_{up} when $\alpha \neq 0$.

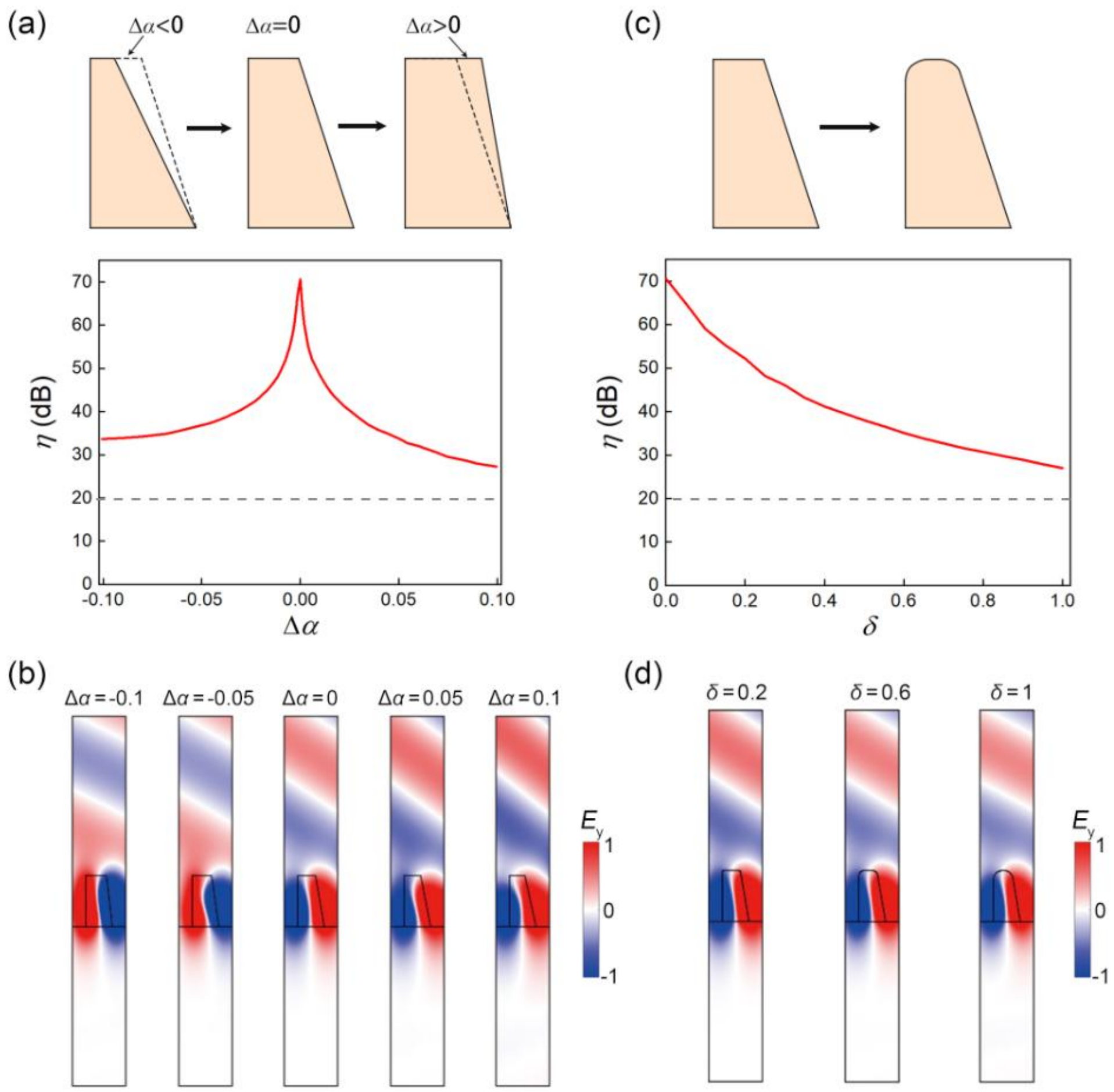

Figure S3 (a) Evolution of radiation asymmetry ratio η with deviation of α from the designed value, showing that the high efficiency is maintained around. The upper panel illustrates the structural variations. (b) Corresponding electrical field distributions for different $\Delta\alpha$, confirming the preservation of the quasi-UGR mode. (c) Evolution of η with increasing corner rounding parameter δ. $\delta = 2r/[w(1-\alpha)]$, where r denotes the corner rounding radius. The upper panel shows the rounded corner geometry. (d) Electrical field distributions at different δ, demonstrating that the quasi-UGR modes remain stable under moderate etching-induced imperfections. The dashed line indicates the 20 dB threshold for strong unidirectional radiation. In both scenarios, the initial value of α is 0.325. Even with these imperfections, the quasi-UGR mode remains robust, and the high radiation asymmetry ratio η (well above the 20 dB threshold) is maintained. The field distributions further verify that the unidirectional radiation capability is well maintained.

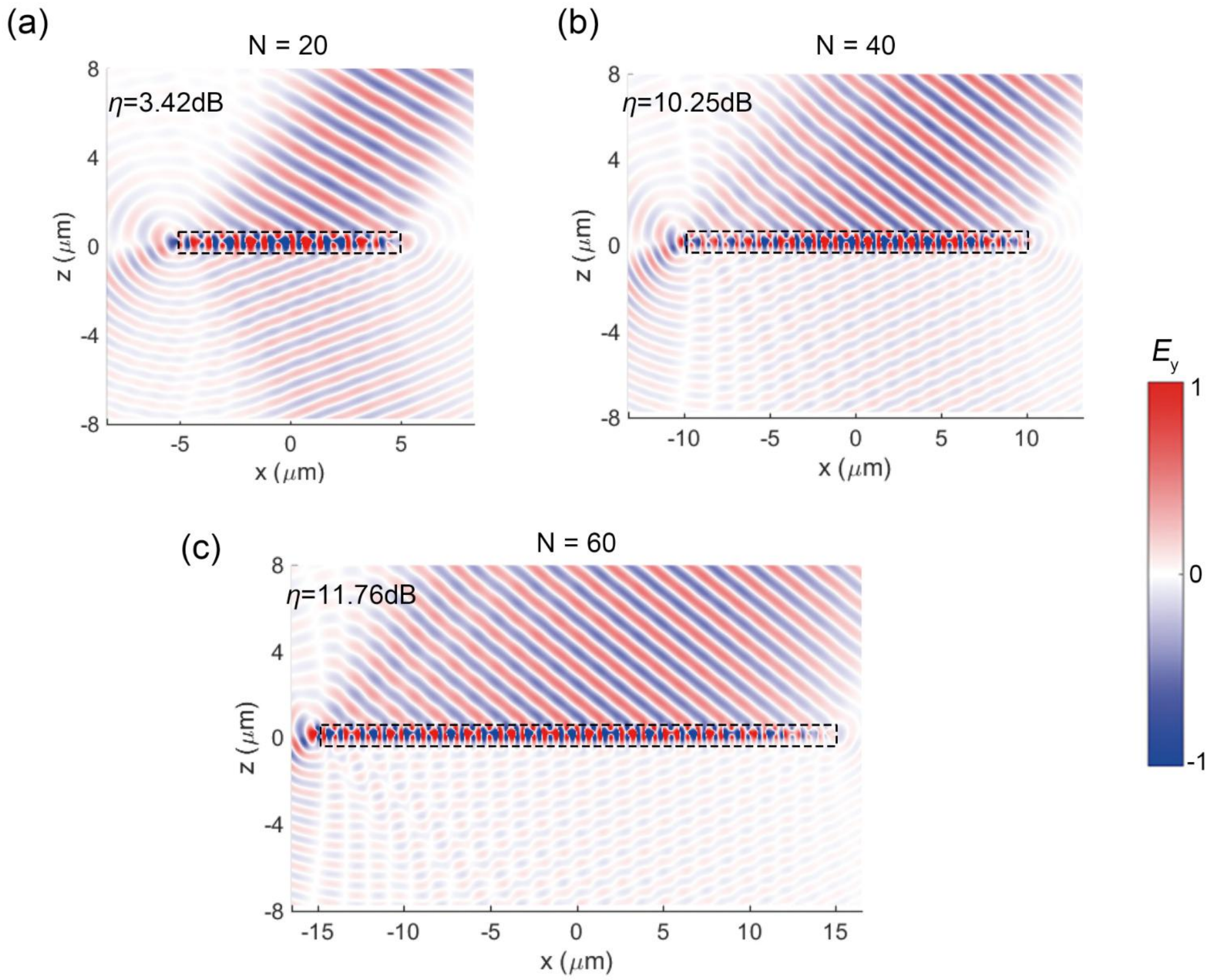

Figure S4 Simulated electric field profile E_y of quasi-UGR mode for infinite arrays of period number N = 20, 40, and 60. The asymmetry parameter α is fixed as 0.325, and all other parameters of the photonic crystal slab keep the same with the case in Fig.2 of the main text. The photonic crystal slabs are marked by black dashed boxes. The electric field profiles show that the radiation asymmetry ratio η grows from 3.42 dB to 11.76 dB when N is increased from 20 to 60. Although the obtained η values are lower than the results of ideal periodic structures, distinct unidirectional radiation characteristics can still be observed. This verifies that robust unidirectional radiation can be achievable in finite-size photonic crystal slabs.

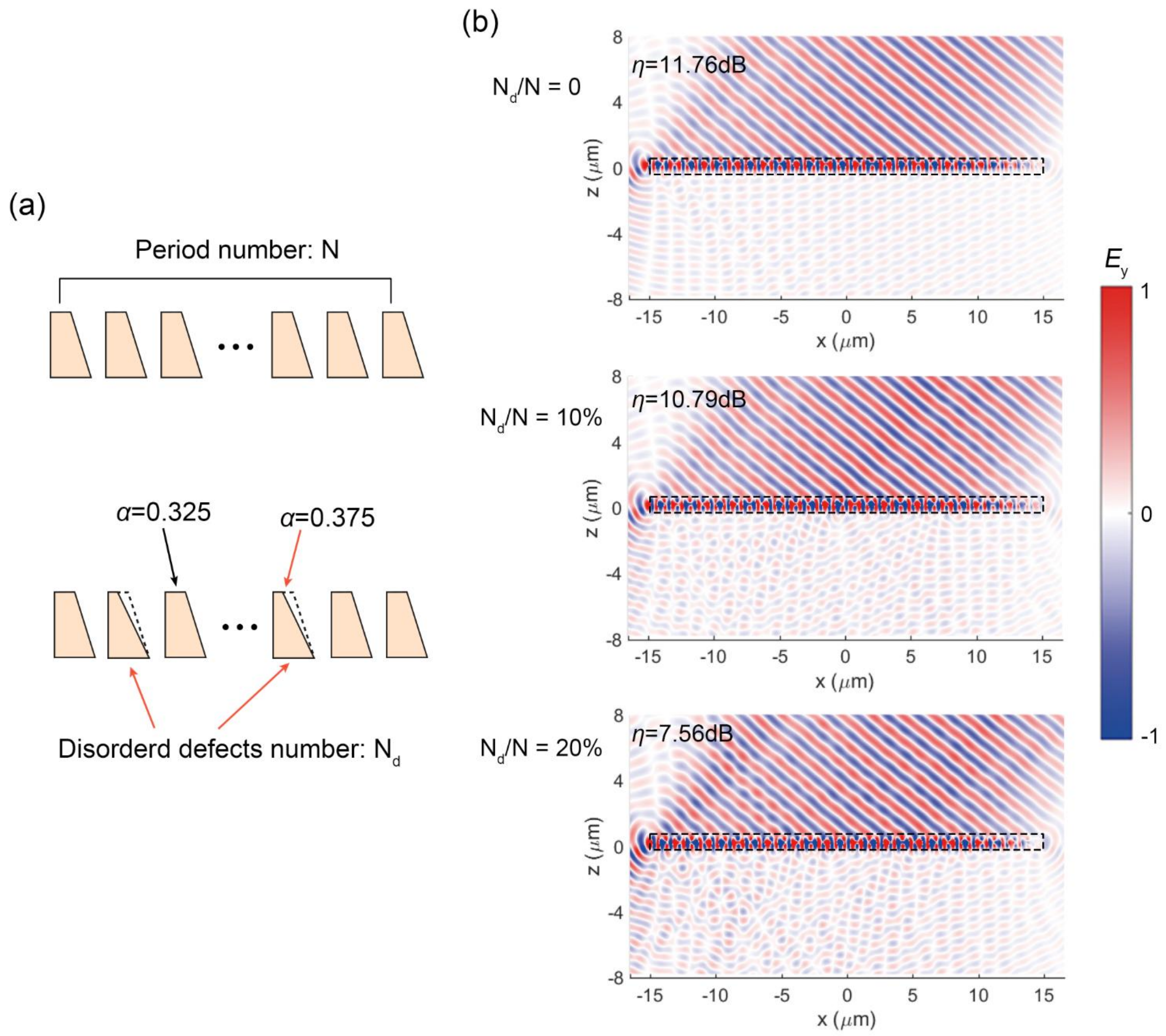

Figure S5 (a) Finite-size photonic crystal slabs without and with disordered defects. The period number N is fixed at 60, and the asymmetry parameter α of defective structures is set to 0.375. N_d represents the number of defective units. All other structural parameters remain consistent with those in Fig. 2 of the main text. (b) Electric field profiles as N_d/N increases from 0 to 20%. Black dashed boxes outline the photonic crystal slabs. When N_d/N is increased from 0 to 20%, the radiation asymmetry ratio η decreases gradually. Nevertheless, the electric field profiles confirm that unidirectional radiation characteristics remain well preserved. The results prove that the proposed quasi-UGRs can still achieve effective unidirectional radiation performance under a certain level of disordered defects.
